# In Vitro Effects of Acitretin on Human Neuronal SH-SY5Y Cells

**DOI:** 10.1007/s11064-022-03716-8

**Published:** 2022-08-20

**Authors:** Aojie Cai, Nana Liu, Zehong Lin, Xiao Li, Jingmin Wang, Ye Wu, Kai Gao, Yuwu Jiang

**Affiliations:** 1 Department of Pediatrics, Peking University First Hospital, No.1 Xi’an Men Street, West District, Beijing, 100034 China; 2 Beijing Key Laboratory of Molecular Diagnosis and Study on Pediatric Genetic Diseases, Beijing, China; 3 Children Epilepsy Center, Peking University First Hospital, Beijing, China; 4 Key Laboratory for Neuroscience, Ministry of Education/National Health and Family Planning Commission, Peking University, Beijing, China; 5 Center of Epilepsy, Beijing Institute for Brain Disorders, Beijing, China

**Keywords:** Acitretin, SH-SY5Y cell, Neuronal differentiation, RNA-seq, Neurons

## Abstract

Acitretin is an oral drug approved by the Food and Drug Administration that is commonly used to treat psoriasis. In recent years, acitretin has been identified as a candidate drug for the treatment of Alzheimer’s disease, but its role in neuronal development is still unclear. In this study, the human neuroblastoma cell line SH-SY5Y was used as a model to study neuronal differentiation. We found that acitretin effectively promoted the differentiation of SH-SY5Y cells into neuronal cells and upregulated the expression of the neuronal marker β-III tubulin and the mature neuronal marker NFH. Differentially expressed genes were identified by RNA sequencing and analyzed by bioinformatics approaches. The results showed that genes associated with neuron development-related pathways, such as *SSPO* and *KCNT1*, had significant changes in expression. Analysis showed that *PRKCA* and *CAMK2B* may play important roles in the process by which acitretin promotes neurodevelopment. Through whole-cell patch clamping and a microelectrode array assay, we found that acitretin-treated neurons generated electrical spikes similar to those generated by mature neurons. This study provided evidence to support an accessible and safe model of neuron-like cells and verified that acitretin can promote the differentiation of neurons and has the potential to treat brain tumors and neurodevelopmental and neurodegenerative diseases.

**Supplementary Information:**

The online version contains supplementary material available at 10.1007/s11064-022-03716-8.

## Introduction

Acitretin (AC, CAS number: 55079-83-9), also named Ro 10-1670, is an oral drug approved by the Food and Drug Administration (FDA) for the treatment of psoriasis that is effective as monotherapy and belongs to a group of drugs known as second-generation retinoids. Soriatane and Neotigason are brand names of acitretin and are used to treat psoriasis [1, 2]. The natural and synthetic compounds of acitretin have activity similar to that of vitamin A; can affect the immune system; regulate cell growth, differentiation, and proliferation; and play a role in embryonic development. Acitretin also induces anti-inflammatory [3] and antineoplastic responses [4] and can inhibit the expression of proinflammatory cytokines such as interleukin-6 (IL-6), migration inhibitory factor-related protein 8 (MRP-8) and interferon-gamma. It also carries the risks of teratogenicity and causing hepatitis. Acitretin has been used in the treatment of skin diseases in previous studies, but recent studies have found that acitretin could be a candidate drug for the treatment of Alzheimer’s disease (AD). Phase II clinical trials of acitretin in treating AD have been completed (NCT01078168) [5, 6]. However, acitretin’s effect on neuronal development is not clear.

The SH-SY5Y cell line was established in 1978 and is a subclone of the human neuroblastoma cell line SK-N-SH [7]. Normal SH-SY5Y cells appear to be nonpolarized, with short processes and the expression of immature neuronal markers [8], but some differentiation-inducing factors, such as all-trans retinoic acid (RA), 12-0-tetradecanoylphorbol-13-acetate (TPA), and cholesterol, can induce SH-SY5Y cells to differentiate into cells with neuronal morphology [9] and express markers of mature neurons, such as GAP-43, NeuN, SV2, NSE, SYN, and MAP. Thus, the SH-SY5Y cell line is a classic and widely accepted cell model for the study of neuronal development [10, 11].

Acitretin activates nuclear retinoic acid receptors (RARs), resulting in the induction of cell differentiation, inhibition of cell proliferation, and inhibition of tissue infiltration by inflammatory cells [12]. A recent study showed that acitretin can increase the number of excitatory synapses in AD mice and increase the expression of neuronal and glial markers in the visual cortex [6], which suggests that it may have a pro-neuronal differentiation effect. However, the effect of acitretin on neuronal differentiation is still unclear. In this study, we found that acitretin promoted the differentiation of neuroblastoma SH-SY5Y cells into functional excitatory neurons and upregulated the expression of the neuronal markers NFH and β-tubulin III. These findings reveal a safe method for SH-SY5Y cell differentiation and highlight an important role of acitretin in the process of neuronal differentiation.

## Methods

### Cell Culture and Neuronal Differentiation of SH-SY5Y Cells

SH-SY5Y cells were cultured in DMEM/F12 medium (Gibco, USA) supplemented with 10% (v/v) heat-inactivated fetal bovine serum (FBS) (Gibco, USA) and a penicillin–streptomycin mixture (1:100; Gibco). All cultures were incubated in a CO_2_ incubator (Thermo Fisher, USA) at 37 °C with 5% CO_2_ (v/v) and a humidity of 95%. The culture medium was changed twice a week. Cultures at 70–80% confluence were used for passaging for experiments. The cells were divided into a total of four groups. Cells in the untreated group (control), 0.1% DMSO group, 10 μM RA (Sigma, USA) and 10 μM AC (Sigma, USA) groups were differentiated for 4 days and 7 days. The medium and drugs were replaced with fresh medium and drugs once every two days.

### Immunofluorescence

On the 4th day of differentiation in culture, cells cultured on microscope coverslips were fixed with 4% paraformaldehyde (PFA) for 20 min before being permeabilized with 0.3% Triton X-100 for 15 min. The coverslips were then blocked with 3% bovine serum albumin (BSA) in PBS for 1 h at room temperature and incubated with rabbit anti-β-tubulin III (1:400, Cell Signaling Technology, USA) and mouse anti-neurofilament heavy polypeptide (1:400, Cell Signaling Technology, USA) antibodies overnight at 4 °C. After washing three times with PBS, the coverslips were incubated with Alexa Fluor 488-conjugated goat anti-mouse (1:200, Invitrogen, USA) and Alexa Fluor 488-conjugated goat anti-rabbit (1:200, Invitrogen, USA) secondary antibodies for 1 h at room temperature. Nuclei were stained with 5 μg/ml Hoechst 33,258 (Invitrogen, USA) at room temperature for 15 min. Finally, the coverslips were observed with an FV10i confocal microscope (Olympus, Japan). Fluorescence images of each group were analyzed with ImageJ analysis software (NIH, USA).

### Real-Time PCR

Total RNA was extracted with TRIzol reagent (Life Technologies, USA), the RNA purity and quantity were checked using a NanoDrop 2000 spectrophotometer (Agilent Technologies, Santa Clara, CA, USA), and the RNA integrity was assessed using an Agilent 2100 bioanalyzer. Quantitative PCR was carried out with GoTaq® qPCR Master Mix (Promega, USA) in a 20 μl final volume using a 7500 Real-Time PCR System (Life Technologies, USA) according to the manufacturer’s instructions. Samples were run for 40 cycles under default thermal cycling conditions for SYBR Real-Time PCR (stage 1: 1 cycle, 95 °C for 2 min; stage 2: 40 cycles, 95 °C for 15 s and 60 °C for 1 min). The primer sequences were as follows: *GAPDH* forward, 5′-GTGGACCTGACCTGCCGTCT-3′; *GAPDH* reverse, 5′-GGAGGAGTGGGTGTCGCTGT-3′; *KCNT1* forward, 5′-CCGGACCTTCGAGTTTGACG-3′; *KCNT1* reverse, 5′-GTCGCTCATCTTGAAGCCG-3′; *SSPO* forward, 5′-TCTGTGGGCTCTACAATGGC-3′; and *SSPO* reverse, 5′-GCCAGGTTCTACATCTGCGT-3′. Each sample was analyzed routinely in triplicate. Relative expression levels were calculated using the 2^−ΔΔCT^ method.

### RNA-Seq and Transcriptome Analysis

RNA was extracted from untreated and 10 μM AC-treated SH-SY5Y cells after 4 days of culture, and a total amount of 2 µg RNA per sample was used as input material for RNA sample preparation. Sequencing libraries were prepared by following the VAHTS mRNA-seq v2 Library Prep Kit (Illumina, San Diego, CA, USA) user manual, and index codes were added to attribute sequences to each sample. The libraries were sequenced using the Illumina NovaSeq platform with a paired-end 150 bp dual indexing protocol. Differential expression analysis between the two conditions was performed using the DEGseq R package (1.20.0). Differentially expressed genes were defined as those for which the adjusted P value was less than 0.05 and the log2 (fold change) was greater than 1.

GO functional enrichment and KEGG pathway enrichment analyses were performed with KOBAS 3.0. GO terms associated with the set of intersecting target differentially expressed genes identified by RNA-seq were determined and differential expression analysis was conducted using the LIMMA software package (Version 3.10.3, http://www.bioconductor.org/packages/2.9/bioc/html/limma.html). GO functional enrichment analysis encompasses three independent ontologies: molecular functions, biological pathways and cell components. The KEGG database is a collection of graphical metabolic pathways, including pathways involved in various cell biochemical processes. The Gene Set Function–Functional Enrichment-mRNA Enrichment module of the MATHT tool was selected for gene enrichment analysis, and P < 0.05 was considered the significance threshold. The CyKEGGParser plug-in (Version 1.2.7, http://apps.cytoscape.org/apps/CyKEGGParser) was applied to draw pathway maps for significantly enriched related pathways.

### Western Blot Analysis

On the 4th and 7th days of differentiation, cells in the tissue culture dishes were washed three times in precooled PBS and then incubated in ice-cold RIPA lysis buffer (20 mM Tris–HCl (pH 8.1), 150 mM NaCl, 0.1% NP-40, 1% SDS, 0.5% sodium deoxycholate, 1 mM PMSF and 1 mM protease inhibitor cocktail) for 10 min at 4 °C (100 μl per 35 mm Petri dish). The cells were then centrifuged at 14,000 rpm for 30 min at 4 °C. The supernatant was collected, and the protein concentration was measured using a BCA protein assay kit (Beyotime, China). Then, the supernatant was mixed with loading buffer (5×) and boiled for 10 min at 100 °C. Proteins were separated on 8% SDS polyacrylamide gels according to the molecular weight of the detected proteins and transferred onto PVDF membranes with an iBlot-2 system (25 V, 9 min). The membranes were blocked with 5% nonfat milk in TBST (TBS containing 0.1% Tween 20) for 1 h at RT and incubated with mouse anti-neurofilament heavy polypeptide (1:1000, Cell Signaling Technology, USA) and mouse anti-β-actin (1:1000, Applygene, China) primary antibodies overnight at 4 °C prior to incubation with horseradish peroxidase (HRP)-conjugated goat anti-mouse secondary antibodies (Beyotime, China) for 1 h at RT. Finally, proteins were detected by chemiluminescence. Immunoblotting was conducted in triplicate for every set of experiments. Target protein band densities were normalized to β-actin band densities, and band densities were measured using ImageJ analysis software (NIH, USA).

### Microelectrode Array (MEA) Recording

SH-SY5Y cells treated with AC for 4 days were cultured and induced to differentiate on CMOS-MEA chips for recording of simultaneous spontaneous firing recording. Cells were seeded over the modified electrode surfaces and incubated at 37 °C in 100% air. The spikes in each culture 4 days after AC treatment were monitored for up to 5 min and then analyzed offline with BrainWave 4 analysis software (3Brain, USA).

### Whole-Cell Patch Clamp Recording

AC-treated SH-SY5Y cells were plated on glass coverslips pretreated with 0.1 mg/ml poly-D-lysine. After 10 days, the cells were perfused with external recording solution that contained 145 mM NaCl, 3 mM KCl, 2 mM CaCl_2_·2H_2_O, 2 mM MgCl_2_·6H_2_O, 10 mM HEPES and 10 mM d-glucose, with the pH adjusted to 7.35 with NaOH and osmotic pressure adjusted to 310 mOsm/L with sucrose. The recording electrode resistances were set in the range of 3‐6 MΩ (thin-walled filamented borosilicate glass pipettes, #TW150F-4, World Precision Instruments) filled with an internal pipette solution that contained 135 mM K-glucose, 5 mM KCl, 10 mM HEPES, 5 mM EGTA, 0.5 mM CaCl_2_·2H_2_O, 2 mM MgCl_2_·6H_2_O, 5 mM ATP and 0.3 mM Na-GTP (pH adjusted to 7.35 with KOH). The current clamp mode was applied to measure the evoked action potentials and firing pattern. The frequency and amplitude of the evoked APs were examined offline with Clampfit 10.1 (Axon Instruments) and Igor (WaveMetrics, Inc., Portland, OR). All statistical analyses were performed using GraphPad Prism 8.0 software.

### Statistical Analysis

Data are presented as the means ± standard errors of the mean (SEMs). Between‐group differences were analyzed with ordinary one-way ANOVA. A P value < 0.05 was considered statistically significant.

## Results

### Acitretin Promotes the Differentiation of SH-SY5Y Cells into Neuron-Like Cells In Vitro

**Fig. 1:**
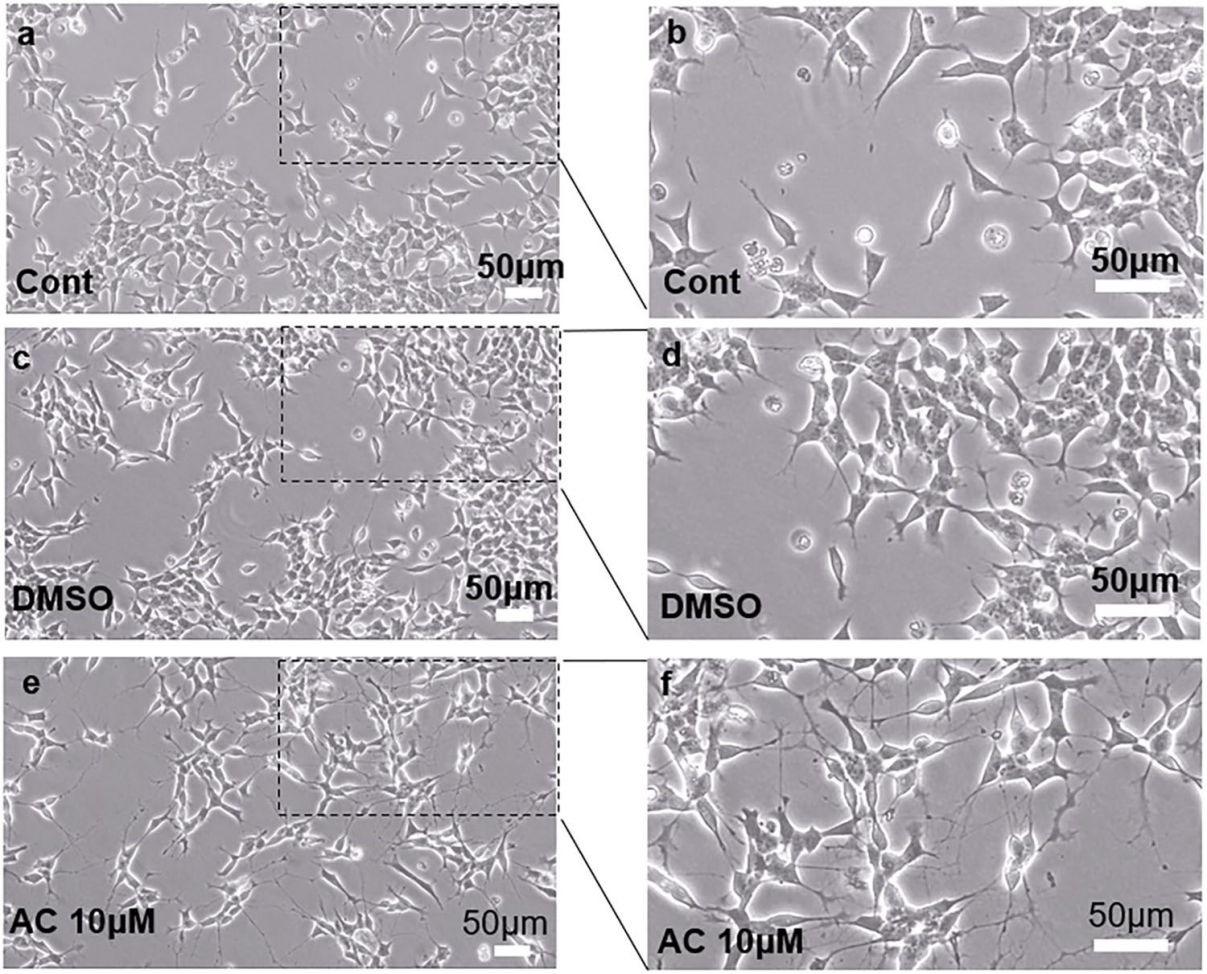
Microscopic observation of SH-SY5Y cells after treatment with 10 µM acitretin for 4 days. Treated cells formed longer projections and produced several branches compared with control cells and DMSO-treated cells. Scale bar = 50 μm

First, we observed the effect of AC on the cell morphology of SH-SY5Y cells. On Day 4, we obtained images of normal SH-SY5Y, 0.1% DMSO-treated SH-SY5Y- and 10 μM AC-treated SH-SY5Y cells by phase contrast microscopy (Figs. 1 and S1). As shown in Fig. 1a and b, the culture of normal undifferentiated flat polygonal SH-SY5Y cells contained several short, swollen cells. Considering that AC is dissolved in DMSO, we used SH-SY5Y cells treated with the same concentration of DMSO as another control group and found that treatment with 0.1% DMSO did not change the morphology of SH-SH5Y cells (Fig. 1c, d). After four days of 10 μM AC treatment, the neurons acquired a rounded morphology, and these long ridges always produced several branches and formed a network (Fig. 1f, e). The morphology of AC-treated SH-SY5Y cells was very similar to that of neurons.

### Acitretin Upregulated the Expression of Neuronal Marker Proteins in SH-SY5Y Cells

**Fig. 2:**
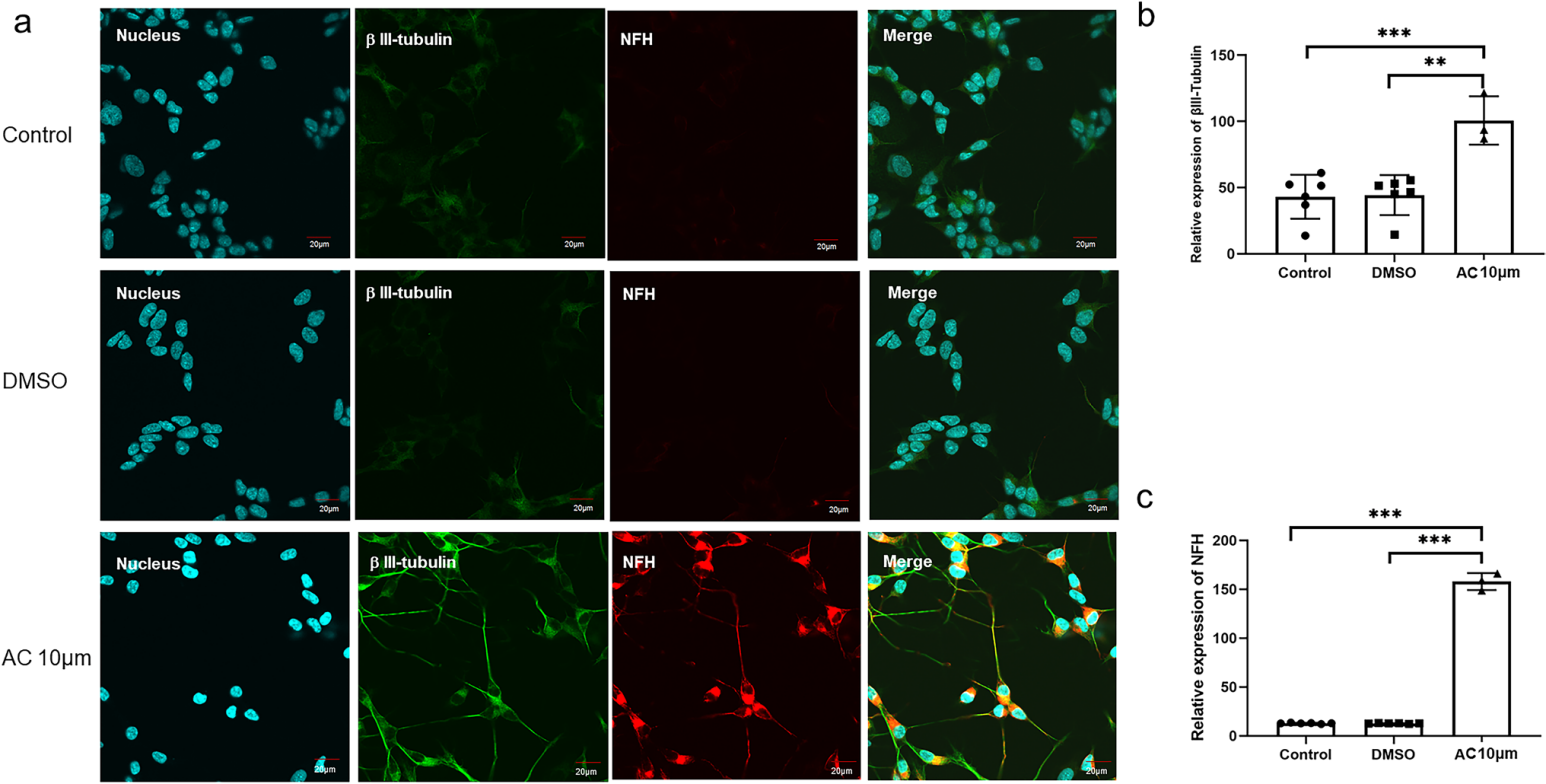
Immunostaining of 10 μM acitretin-treated, DMSO-treated and control SH-SY5Y cells. **a** βIII-Tubulin was used to mark neurons (green), NFH (neurofilament H) was used to mark mature neurons (red), Hoechst 33258 was used to stain nuclei (blue); individual and merged images in the fluorescence channels are shown. Scale bar = 20 μm. **b** and **c** The relative expression of βIII-tubulin (**b**) and NFH (**c**) in the AC-treated (n = 3), DMSO (n = 6) and control groups (n = 6) at 4 days. ***P value < 0.001, **P value < 0.01

To determine the development of SH-SY5Y cells induced by AC to differentiate into neurons, we investigated the expression of neuronal markers by immunofluorescence microscopy, qPCR and WB. We first observed the neuron-specific marker β-tubulin III and the mature neuron marker neurofilament heavy polypeptide (NFH) in cells by immunofluorescence staining. We found that β-tubulin III was significantly immunolabeled in extended neurites and branches and that NFH staining was mainly restricted to cell bodies to form the cytoskeleton but was also observed in some elongated axons (Fig. 2a). After treatment with AC for 4 days, the expression of β-tubulin III and NFH was upregulated (Fig. 2b, c). When AC treatment was continued, we found that NFH protein expression was still significantly increased after 7 days of treatment (Fig. S2), indicating that AC treatment could cause SH-SY5Y cells to enter a stage of gradual differentiation and maturation.

### GO Functional Enrichment and KEGG Pathway Enrichment Analyses

**Fig. 3:**
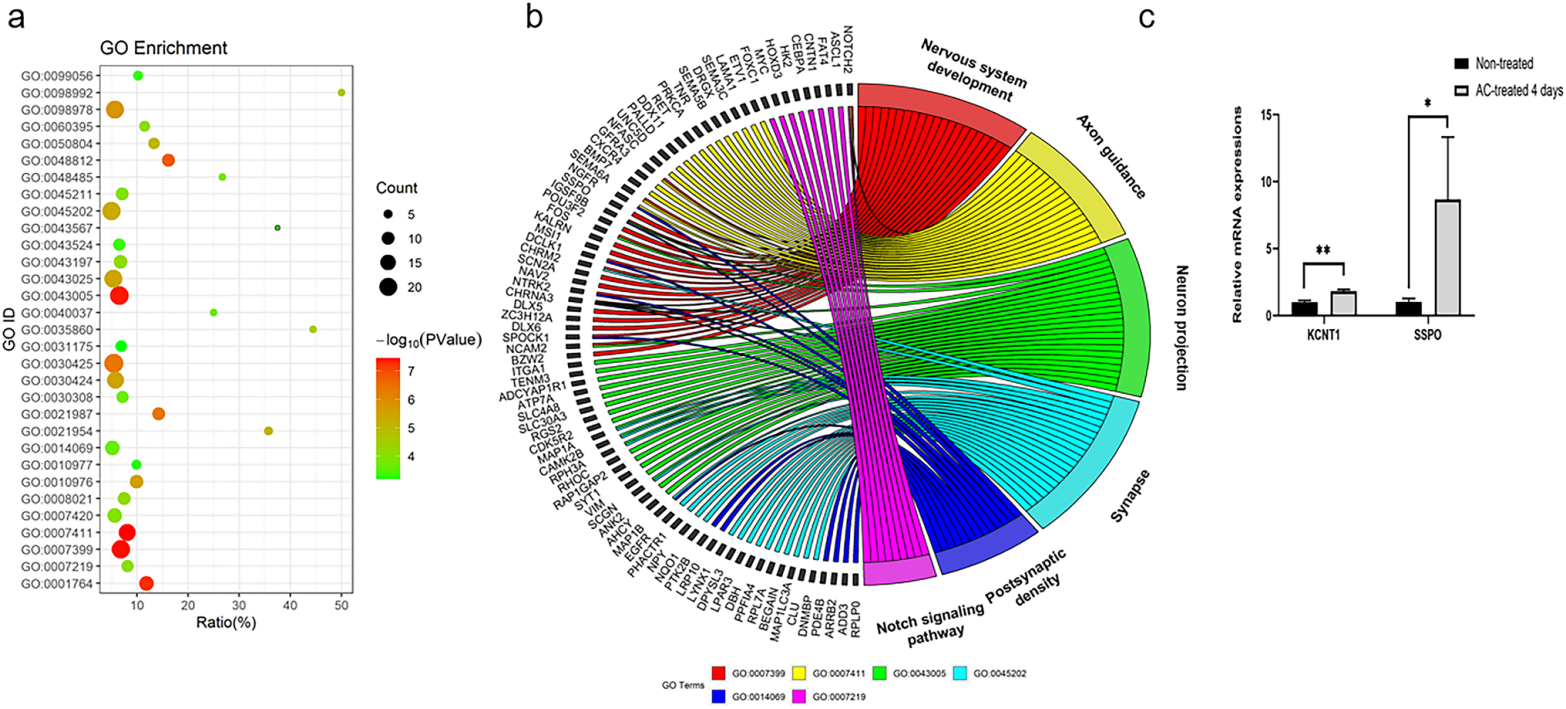
GO term enrichment analysis of DEGs. **a** The bubble plot shows the functional enrichment of 31 neuron-related GO terms. The size of the bubbles in the plot represents the number of enriched DEGs in each pathway, the color of the bubbles represents the P value, the vertical axis shows the relevant GO terms, and the horizontal axis shows the percentages of DEGs among all genes in each pathway. **b** The bar plots show the changes in the expression between control SH-SY5Y cells and SH-SY5Y cells treated with AC for 4 days. **c** The mRNA expression of *KCNT1* and *SSPO* in the AC-treated and untreated groups at 4 days (n = 3). **P value < 0.01, *P value < 0.05

**Fig. 4:**
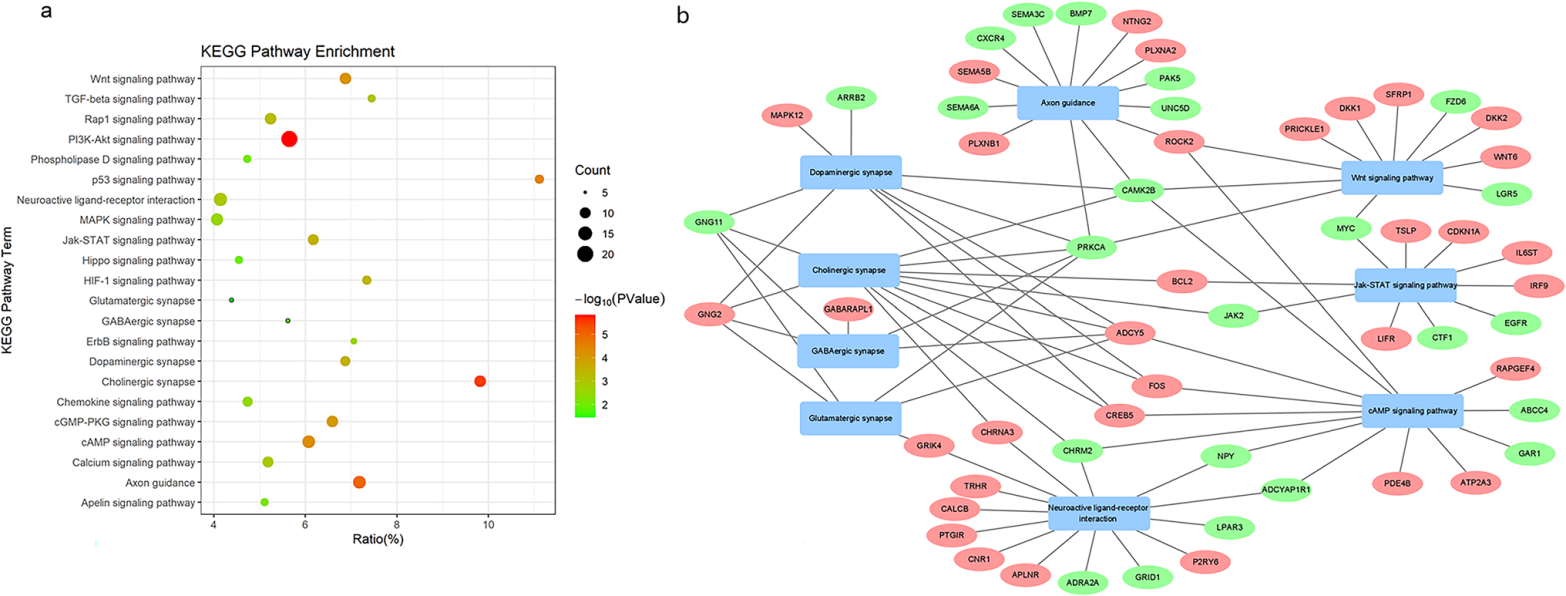
KEGG pathway enrichment analysis of DEGs. **a** The bubble plot shows the functional enrichment of 22 KEGG pathways. The size of the bubbles in the plot represents the number of enriched DEGs in each pathway, the color of the bubbles represents the P value, the vertical axis shows the relevant KEGG pathway, and the horizontal axis shows the percentages of DEGs among all genes in each pathway. **b** The interactions of significantly enriched neuron-related KEGG pathways with DEGs

We next sought to better understand the genetic basis of the neuronal differentiation of SH-SY5Y cells. We performed RNA‐seq analysis on AC-treated differentiated SH-SY5Y cells versus those that were nondifferentiated (Supplementary File S1). The bubble plot shows the results of gene ontology (GO) functional enrichment analysis with SH-SY5Y cell differentiation. GO functional enrichment analysis of differentially expressed genes identified 6 significantly enriched GO terms related to neuronal differentiation, as shown with Circos plots: regulation of nervous system development, regulation of axon guidance, regulation of neuron projection, regulation of synapse, regulation of postsynaptic density and regulation of Notch signaling pathway (Fig. 3a and b, Supplementary File S2). The RNA-seq results showed that the expression of the neuronal ion channel gene *KCNT1* and the neurite outgrowth gene *SSPO* was upregulated, and this finding was verified by qPCR (Fig. 3c). KEGG pathway analysis identified 22 differential pathways associated with the GO terms (Table 1), and we chose the top 9 pathways that were closely associated with the neuron development-related significantly enriched terms to construct a pathway network map and aimed to identify the module showing the synergistic effects of important genes and pathways (Fig. 4a, b). In the interaction network of the top 9 altered pathways, which contained 57 nodes and 91 interrelationships, the neuroactive ligand–receptor interaction signaling pathway was the most important pathway with the highest interaction degree (upregulation degree = 8, downregulation degree = 6, and total degree = 14). The most important gene was *PRKCA*, which was at the center of the altered pathway interaction network and is a key player in the induction of cellular responses to various ligand–receptor systems and external stimuli. *CAMK2B* also plays an important role in the progression of AC-treated SH-SY5Y cell differentiation and has critical roles in synaptic plasticity, long-term potentiation and neurotransmitter release.

**Table 1:** KEGG pathway enrichment results of up/down-regulated differential genes

KEGG_Term	Count	P value	Gene	
			Up-degree	Down-degree
Neuroactive ligand-receptor interaction	14	0.00108716	CNR1, GRIK4, PTGIR, TRHR, CHRNA3, APLNR, CALCB, P2RY6	CHRM2, NPY, ADCYAP1R1, LPAR3, GRID1, ADRA2A
Axon guidance	13	9.47E − 06	PLXNB1, SEMA5B, NTNG2, PRKCA, PLXNA2, CAMK2B, ROCK2	BMP7, SEMA3C, CXCR4, SEMA6A, PAK5, UNC5D
cAMP signaling pathway	13	4.94E − 05	ADCY5, FOS, CREB5, ATP2A3, RAPGEF4, PDE4B, ROCK2	NPY, ADCYAP1R1, CHRM2, ABCC4, GAR1, CAMK2B
Wnt signaling pathway	11	6.59E − 05	PRICKLE1, SFRP1, DKK2, WNT6, ROCK2, DKK1	LGR5, FZD6, MYC, PRKCA, CAMK2B
Cholinergic synapse	11	2.95E − 06	ADCY5, FOS, CREB5, CHRNA3, GNG2, BCL2	PRKCA, CAMK2B, GNG11, JAK2, CHRM2
Jak-STAT signaling pathway	10	0.000317692	IRF9, IL6ST, LIFR, TSLP, CDKN1A, BCL2	EGFR, CTF1, JAK2, MYC
Dopaminergic synapse	9	0.000300609	ADCY5, FOS, CREB5, GNG2, MAPK12	PRKCA, CAMK2B, GNG11, ARRB2
Glutamatergic synapse	5	0.03522864	GRIK4, ADCY5, GNG2	PRKCA, GNG11
GABAergic synapse	5	0.014329512	ADCY5, GABARAPL1, GNG2	PRKCA, GNG11
PI3K-Akt signaling pathway	20	1.47E − 06	SGK1, TNR, ITGA1, CREB5, NTRK2, NGFR, GNG2, VTN, CDKN1A, BCL2	LAMA1, COL4A2, PRKCA, EGFR, CHRM2, GNG11, JAK2, COL4A1, LPAR3, MYC,
p53 signaling pathway	8	2.96E − 05	SESN3, CDKN1A, CD82, RRM2B, BCL2	CCNB2, IGFBP3, RRM2
cGMP-PKG signaling pathway	11	9.44E − 05	ADCY5, MRVI1, ATP2A3, CREB5, ROCK2, RGS2, PDE5A	KCNMA1, ADRA2A, PPIF, GAR1
HIF-1 signaling pathway	8	0.000427	CDKN1A, BCL2	PRKCA, EGFR, SLC2A1, PFKP, CAMK2B, LDHA,
Rap1 signaling pathway	11	0.000608	CNR1, ADCY5, RAPGEF4, NGFR, MAPK12, SIPA1,	PRKCA, EGFR, ID1, LPAR3, SIPA1L2
TGF-beta signaling pathway	7	0.000894	TGFB1, GREM2, SMAD9,	BMP7, ID4, ID1, MYC
Calcium signaling pathway	10	0.001147	ORAI3, ATP2A3, TRHR,	PRKCA, EGFR, CAMK2B, PTK2B, CXCR4, PPIF, CHRM2
ErbB signaling pathway	6	0.002657	CDKN1A	PRKCA, EGFR, CAMK2B, PAK5, MYC
MAPK signaling pathway	12	0.002766	TGFB1, FLNC, FOS, NTRK2, NGFR, MAPK12, DUSP16, TAOK1	PRKCA, EGFR, ARRB2, MYC,
Chemokine signaling pathway	9	0.003532	ADCY5, ROCK2, GNG2	ARRB2, GRK4, GNG11, PTK2B, JAK2, CXCR4
Apelin signaling pathway	7	0.006569	PLAT, ADCY5, APLNR, EGR1, GNG2, GABARAPL1	GNG11
Phospholipase D signaling pathway	7	0.009658	ADCY5, RAPGEF4	PLPP3, PRKCA, EGFR, PTK2B, LPAR3
Hippo signaling pathway	7	0.011733	TGFB1, FZD6, WNT6	BMP7, ID1, MYC, TEAD4

### Acitretin Induced the Differentiation of SH-SY5Y Cells into Excitable Neurons

**Fig. 5:**
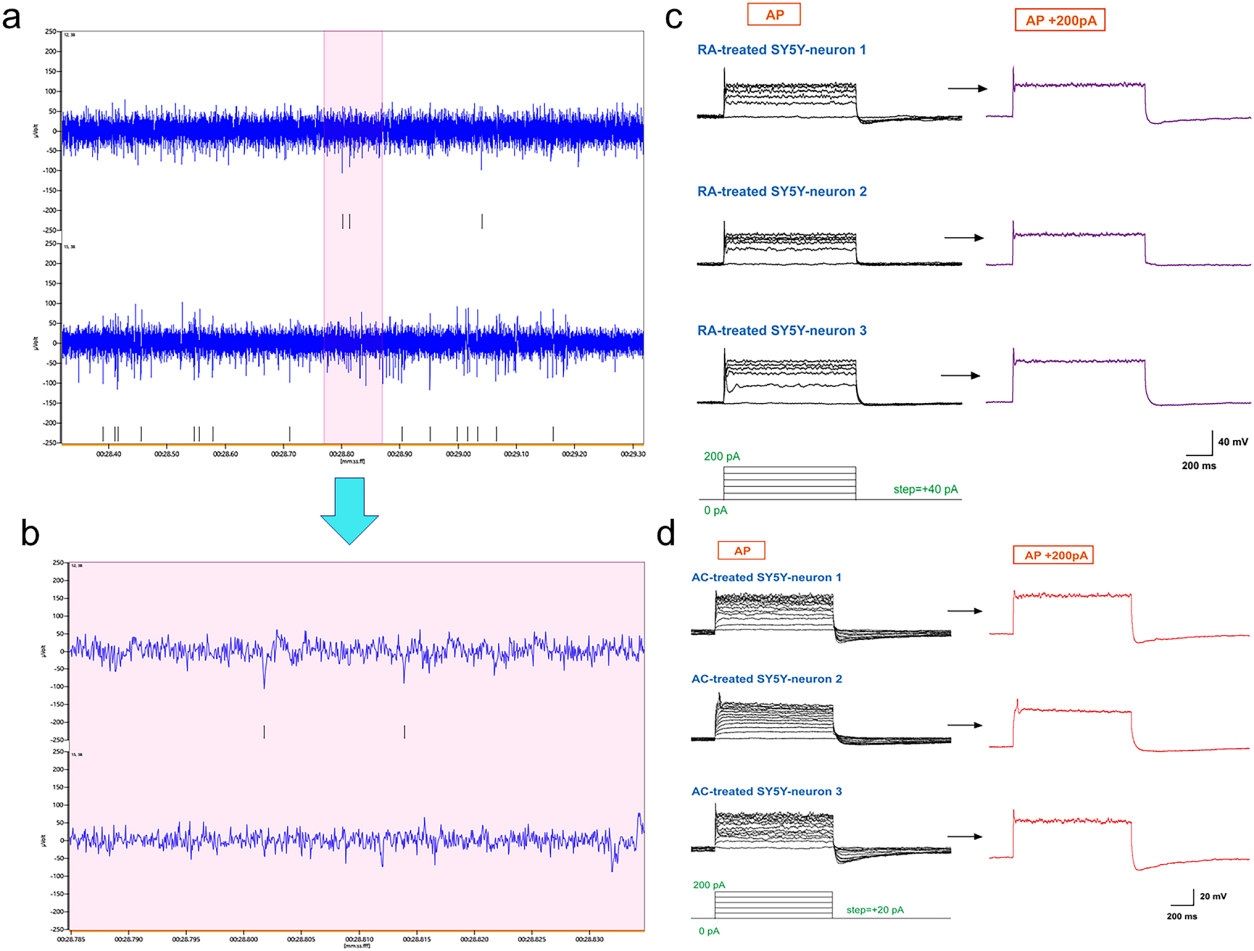
MEA assay and whole-cell patch clamping of AC-induced neurons. **a** Recording of field potential changes over approximately 0.5 s. **b** A magnified image of the marked area. **c** and **d** Example traces of evoked APs in RA-treated (**c**) and AC-treated SH-SY5Y (**d**) neurons in response to test currents ranging from 0 to 200 pA with a step of 20 pA and duration of 1 s. All measured cells showed a single firing pattern at high injected currents. **c** The scale bars represent 200 ms (x‐axis) and 40 mV (y‐axis). **d** The scale bars represent 200 ms (x‐axis) and 20 mV (y‐axis)

To verify that AC-induced SH-SY5Y neurons are electrophysiologically active neurons, we used the MEA technique to measure the field potential of SH-SY5Y cells treated with AC for 4 days. The electrode chip of the 3Brain MEA probe has a high density and can detect single cells. Regarding the electrophysiological activity, we found that spikes appeared continuously on some electrodes (Fig. 5a, b). After treatment with high-potassium medium, the frequency of the spikes increased. These results showed that AC can induce SH-SY5Y cells to differentiate into functional neurons. We also measured the evoked AP firing activity of AC-treated SH-SY5Y neurons by whole-cell patch clamping. Some of the AC-treated SH-SY5Y neuron-like cells showed excitability at an injected current of 60 pA, and the cells always showed a single spike under injection of high currents and exhibited the same firing pattern and amplitude as RA-treated SH-SY5Y neuron-like cells (Fig. 5c, d).

## Discussion

In traditional research, RA is the most commonly used treatment to induce SH-SY5Y cells to differentiate into neuron-like cells. In this study, we first proposed a method for AC-induced neuron-like differentiation of SH-SY5Y cells. This method produces a homogeneous population of neuronal cells with firing function. When AC is applied, SH-SY5Y cells withdraw from the original cell cycle and begin to differentiate and express many typical neuronal markers, such as β-tubulin III and NFH, and acquire a phenotype and function very similar to those of primary neurons.

In this study, we compared SH-SY5Y cells treated with AC and DMSO for 4 and 7 days with untreated and DMSO-treated SH-SY5Y cells. After induction by AC treatment, the mRNA and protein expression levels were measured, and we found that the expression of neuronal markers and proteins that maintain the normal growth and function of neuronal cells increased; neuronal surface markers were also detected by cellular immunofluorescence staining. After 4 days of AC treatment, RNA was extracted from cells for RNA-seq analysis, and GO functional enrichment analysis and KEGG pathway enrichment analysis were performed with the identified differentially expressed genes. After AC treatment, the mRNA expression of many genes related to the neuronal differentiation process—for example, genes involved in neural development, axon guidance, neuron projection, and synapse formation—was increased, which indicated that after AC treatment, cells are directed toward neuronal differentiation. After analyzing the GO dataset related to neuronal differentiation through KEGG pathway analysis, it was found that many pathways involved in neuronal cell growth and differentiation and intercellular signal transduction, such as the neuroactive ligand–receptor interaction pathway, axon guidance pathway and cAMP signaling pathway, were activated for rapid cell responses. In these pathways, *PRKCA* and *CAMK2B* are the two most important genes, and their expression decreases during the differentiation process. *PRKCA* encodes an important protein in the PKC family, PKC-α, which can regulate the response of cells to a variety of ligand–receptor systems and external stimuli. Studies have found that excessive PKC activation can inhibit prefrontal cortical cognitive function in rats and lead to abnormal neuronal signal transduction [13]. In addition, PKC can regulate the subcellular localization and function of Kras, which associates with Bclxl, thereby inducing apoptosis [14]. *CAMK2B* is an important kinase in the central nervous system and may play a role in long-term enhancement and neurotransmitter release. Thiagarajan et al. found that during the activity of cultured hippocampal neurons from rat pups, the level of *CAMK2B* increased with inhibition of activity, which was consistent with the results of this study [15].

In terms of neuronal function, we used the MEA and whole-cell patch clamping approaches to confirm that induced neuronal cells can produce electric discharges under external stimulation, which provides a simple method and an important cell model for the study of neuronal electrophysiology in neurological diseases, including epilepsy. In the last 20 years, many causative genes and numerous associated variants have been described and considered to be responsible for the corresponding disease, especially for neurodevelopmental disorders and epilepsy. Determining the functional consequences of these mutations often involves studies in in vivo mammalian models and in vitro primary cell models or specific functional cell lines, but disease manifestations in humans cannot be completely faithfully reproduced in the conventional mammalian models, and generating specific functional cell lines can take much time and effort. Here, we generated an AC-treated neuron-like cell line that can not only mimic the differentiation and development of neuronal cells but also be used in electrophysiological and synapse-related research, providing an important and accessible cell model for human neuroscience research.

Acitretin is a RAR/RXR agonist that can promote cell differentiation. Since its discovery, it has been mainly used as a treatment for skin diseases, and its application in the nervous system has not progressed sufficiently. In 2010, a study found that AC can effectively cross the blood–brain barrier [16] and promote the expression of amyloid beta protein precursor (AβPP) and ADAM10 (a disintegrin and metalloproteinase 10), thereby driving the nonamyloidogenic pathway in neuroblastoma cells; in addition, it reduces the Aβ level in APP/PS-1 AD model mice [17] and it is thus regarded as one of the possible treatments for AD. In addition, the enzyme can mediate synaptic forward trafficking, and its active localization in synapses can regulate synaptic functions [18], which may be one of the important mechanisms by which AC can promote the differentiation of SH-SY5Y cells into neurons. A 2014 study evaluated oral acitretin therapy for patients with mild to moderate AD. After treatment, an increase in the level of α-secretase-derived AβPP (AβPP-α) was detected in cerebrospinal fluid (CSF). AβPP processing by the nonamyloidogenic pathway also seemed to be affected [19, 20]. Since then, the study of AC in the treatment of AD has progressed slowly. In 2021, a study reported that in an AD mouse model, the behavioral deficits and the increase in excitatory synapse numbers in layers II/III were reversed after treatment with AC, which corrected the aberrant activity of local functional networks [6]. Successful studies in animal models have, importantly, promoted the study of the mechanism of AC in neurological diseases. Coincidentally, RA has always been regarded as one of the drugs for AD treatment; it has long-term regulatory effects on neuroplasticity and mediates neurogenesis, steady-state plasticity, and the ability to form new neurites and neurite extensions. RA can reduce the neurotoxicity of amyloid β (Aβ), correct the attenuation of retinoic acid signaling in the AD model, and exert anti-inflammatory effects. Compared with ATRA, acitretin is more stable and efficient, and these enhanced properties depend on its molecular structure. In previous functional experimental studies, acitretin also had a higher potency than ATRA [21]. This was also confirmed in this study: in the classical cell model that promotes SH-SY5Y cell differentiation, the usual concentration of ATRA is 10 mM, and in this study, 1 mM acitretin had the same effect on promoting neuron-like cell differentiation.

In summary, this study found that AC can also promote the growth of neurites, similar to RA, showing morphological and biochemical effects on neuronal differentiation. Because of the important role of neuronal differentiation in neurodevelopmental and neurodegenerative diseases, this study not only expands the theoretical basis of the use of acitretin in the treatment of AD but also suggests the possibility that acitretin could be used in the treatment of childhood neurodevelopmental disorders and other neurodegenerative diseases. We anticipate further research on this topic to expand the range of possible applications of AC in clinical and scientific research.

## Supplementary Information

Below is the link to the electronic supplementary material.

Supplementary file1 (RAR 4354 KB)

## Data Availability

The original data presented in the study are included in the article materials, and further inquiries can be directed to the author/corresponding authors.

## References

[1] Heath MS, Sahni DR, Curry ZA, Feldman SR (2018). Pharmacokinetics of tazarotene and acitretin in psoriasis. Expert Opin Drug Metab Toxicol.

[2] Guenther LC, Kunynetz R, Lynde CW, Sibbald RG, Toole J, Vender R, Zip C (2017). Acitretin use in dermatology. J Cutan Med Surg.

[3] Chu S, Michelle L, Ekelem C, Sung CT, Rojek N, Mesinkovska NA (2020). Oral isotretinoin for the treatment of dermatologic conditions other than acne: a systematic review and discussion of future directions. Arch Dermatol Res.

[4] Niles RM (2002). The use of retinoids in the prevention and treatment of skin cancer. Expert Opin Pharmacother.

[5] Freese C, Reinhardt S, Hefner G, Unger RE, Kirkpatrick CJ, Endres K (2014). A novel blood-brain barrier co-culture system for drug targeting of Alzheimer’s disease: establishment by using acitretin as a model drug. PLoS ONE.

[6] Rosales Jubal E, Schwalm M, Dos Santos GM, Schuck F, Reinhardt S, Tose A, Barger Z, Roesler MK, Ruffini N, Wierczeiko A, Schmeisser MJ, Schmitt U, Endres K, Stroh A (2021). Acitretin reverses early functional network degradation in a mouse model of familial Alzheimer’s disease. Sci Rep.

[7] Biedler JL, Roffler-Tarlov S, Schachner M, Freedman LS (1978). Multiple neurotransmitter synthesis by human neuroblastoma cell lines and clones. Cancer Res.

[8] Shipley MM, Mangold CA, Szpara ML (2016). Differentiation of the SH-SY5Y human neuroblastoma cell line. J Vis Exp.

[9] Teppola H, Sarkanen JR, Jalonen TO, Linne ML (2016). Morphological differentiation towards neuronal phenotype of SH-SY5Y neuroblastoma cells by estradiol, retinoic acid and cholesterol. Neurochem Res.

[10] Agholme L, Lindström T, Kågedal K, Marcusson J, Hallbeck M (2010). An in vitro model for neuroscience: differentiation of SH-SY5Y cells into cells with morphological and biochemical characteristics of mature neurons. J Alzheimers Dis.

[11] Xicoy H, Wieringa B, Martens GJ (2017). The SH-SY5Y cell line in Parkinson’s disease research: a systematic review. Mol Neurodegener.

[12] Janesick A, Wu SC, Blumberg B (2015). Retinoic acid signaling and neuronal differentiation. Cell Mol Life Sci.

[13] Birnbaum SG, Yuan PX, Wang M, Vijayraghavan S, Bloom AK, Davis DJ, Gobeske KT, Sweatt JD, Manji HK, Arnsten AF (2004). Protein kinase C overactivity impairs prefrontal cortical regulation of working memory. Science.

[14] Bivona TG, Quatela SE, Bodemann BO, Ahearn IM, Soskis MJ, Mor A, Miura J, Wiener HH, Wright L, Saba SG, Yim D, Fein A, Pérez de Castro I, Li C, Thompson CB, Cox AD, Philips MR (2006). PKC regulates a farnesyl-electrostatic switch on K-Ras that promotes its association with Bcl-XL on mitochondria and induces apoptosis. Mol Cel.

[15] Thiagarajan TC, Piedras-Renteria ES, Tsien RW (2002). Alpha- and betaCaMKII. Inverse regulation by neuronal activity and opposing effects on synaptic strength. Neuron.

[16] Holthoewer D, Endres K, Schuck F, Hiemke C, Schmitt U, Fahrenholz F (2012). Acitretin, an enhancer of alpha-secretase expression, crosses the blood-brain barrier and is not eliminated by P-glycoprotein. Neurodegener Dis.

[17] Tippmann F, Hundt J, Schneider A, Endres K, Fahrenholz F (2009). Up-regulation of the alpha-secretase ADAM10 by retinoic acid receptors and acitretin. FASEB J.

[18] Musardo S, Marcello E, Gardoni F, Di Luca M (2014). ADAM10 in synaptic physiology and pathology. Neurodegener Dis.

[19] Endres K, Fahrenholz F, Lotz J, Hiemke C, Teipel S, Lieb K, Tüscher O, Fellgiebel A (2014). Increased CSF APPs-alpha levels in patients with Alzheimer disease treated with acitretin. Neurology.

[20] Khatib T, Chisholm DR, Whiting A, Platt B, McCaffery P (2020). Decay in retinoic acid signaling in varied models of Alzheimer’s disease and in-vitro test of novel retinoic acid receptor ligands (RAR-Ms) to regulate protective genes. J Alzheimers Dis.

[21] Ran L, Tan W, Tan S, Zhang R, Wang W, Zeng W (2005). Effects of ATRA, acitretin and tazarotene on growth and apoptosis of Tca8113 cells. J Huazhong Univ Sci Technolog Med Sci.

